# Neuroprotective Effects of Lipoxin A4 in Central Nervous System Pathologies

**DOI:** 10.1155/2014/316204

**Published:** 2014-09-09

**Authors:** Alessandra Cadete Martini, Stefânia Forner, Allisson Freire Bento, Giles Alexander Rae

**Affiliations:** ^1^Departmento de Farmacologia, Centro de Ciências Biológicas, Universidade Federal de Santa Catarina (UFSC), Campus Universitário, Trindade, 88049-900 Florianópolis, SC, Brazil; ^2^Centro de Inovação e Ensaios Pré-Clínicos (CIEnP), Av. Luiz Boiteux Piazza, 1302-Canasvieiras, 88056-000 Florianópolis, SC, Brazil

## Abstract

Many diseases of the central nervous system are characterized and sometimes worsened by an intense inflammatory response in the affected tissue. It is now accepted that resolution of inflammation is an active process mediated by a group of mediators that can act in synchrony to switch the phenotype of cells, from a proinflammatory one to another that favors the return to homeostasis. This new genus of proresolving mediators includes resolvins, protectins, maresins, and lipoxins, the first to be discovered. In this short review we provide an overview of current knowledge into the cellular and molecular interactions of lipoxins in diseases of the central nervous system in which they appear to facilitate the resolution of inflammation, thus exerting a neuroprotective action.

## 1. Introduction

Neurological diseases, such as Alzheimer's disease, Parkinson's disease, traumatic brain injury, and stroke, among others, as well as conditions leading to chronic neuropathic pain, typically present marked transient or continued neuroinflammation. Whether this inflammatory state has beneficial or detrimental effects is still controversial. Orchestrated actions of microglia, macrophages, and lymphocytes result in a protective mechanism to isolate the damaged brain tissue and destroy the affected cells. Thus, inflammatory responses generally result in a self-limiting healing process. However, if this response is not adequately controlled, the immune system begins to attack previously undamaged cells, which may cause a progressive neuronal loss, amongst many other detrimental effects [1].

Many studies have raised the question that the beneficial effects of diet supplementation with omega-3 (*ω*-3) polyunsaturated fatty acids (PUFAs) could be the result of their metabolism into potentially anti-inflammatory substances [2–5]. Indeed, a growing body of evidence indicates that inflammation may be modulated by endogenously produced lipids that actively participate in dampening host responses to injury, leading to active resolution of the inflammatory process [6]. This group of endogenous proresolving lipid mediators currently comprises lipoxins (LXs), resolvins, protectins, and maresins, all of which have the potential to actively resolve inflammation by signaling metabolic, cellular, and tissue events to return to homeostasis after inflammation, in a process known as catabasis [7].

**Figure 1:**
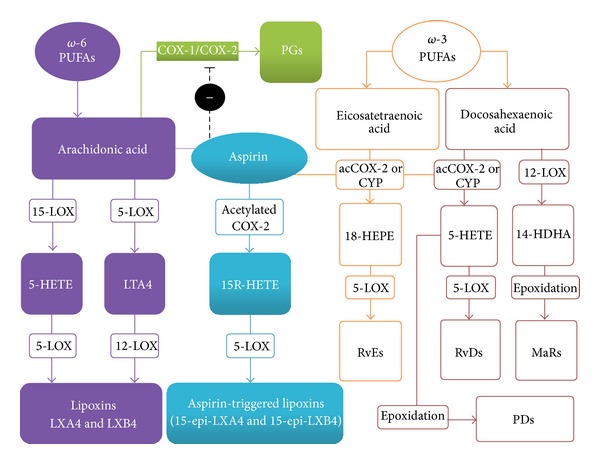
Schematic representation of the main biochemical pathways that mediate the production of proresolution lipid mediators. Arachidonic acid is derived from omega (*ω*)-6 and can be converted into lipoxins by lipoxygenases action. Omega (*ω*)-3 originates EPA-derived resolvins series E and DHA-derived resolvins series D, protectins, and maresins. COX: cycloxygenase; LOX: lipoxygenase; HETE: eicosatetraenoic acid; acCOX-2: acetylated cyclooxygenase-2; CYP: cytochrome P450; LXA4: lipoxin A4; RvEs: resolvins series E; RvD: resolvins series D; MaRs: maresins; PDs: protectins.

All known proresolving lipid mediators are synthetized from PUFAs. Whereas the starting point for synthesis of LXs is arachidonic acid (AA), a *ω*-6 PUFA generated from linoleic acid, resolvins and protectins are products originated from the *ω*-3 PUFAs, eicosapentaenoic acid (EPA) and docosahexaenoic acid (DHA), respectively [8]. Indeed, the same enzymes that metabolize linoleic acid to AA can also convert *α*-linoleic acid into EPA and DHA. However, as the proportion of AA in inflammatory cell membranes is much higher than those of *ω*-3 PUFAs, substrate availability for metabolism of AA by cyclooxygenase (COX) or lipoxygenase (LOX) isozymes is far greater than is seen regarding the metabolism of EPA and DHA. The only two truly endogenous LXs known, LXA4 and LXB4, are typically formed by transcellular metabolism of AA involving sequential LOX activity [9]. In one of these pathways, AA is oxygenated by 15-LOX to generate 15S-HETE, which is then modified by 5-LOX to originate both LXs. Another 2-step pathway for LXA4 and LXB4 formation involves the conversion of AA into leukotriene A4 by LOX-5, followed by its metabolism by LOX-12 [7]. Interestingly, the acetylation of COX-2 by aspirin, while inhibiting the synthesis of prostaglandins and thromboxane, favors the generation of 15R-HETE, which can then be converted by LOX-5 to generate the aspirin-triggered LXs (ATLs) 15-epi-lipoxin A4 and 15-epi-lipoxin B4 [10]. The LXs are subjected to rapid enzymatic breakdown, but ATLs are more resistant to degradation and thus can exert longer-lasting effects. The synthetic pathways of proresolving lipid mediators are depicted in Figure 1, but further details on the synthesis and biological effects of resolvins, protectins, and maresins can be found elsewhere [3, 11, 12].

LXs (and ATLs) promote the majority of their effects by acting on a specific G protein-coupled receptor designated as the ALX/FPR2 receptor, a member of the formyl peptide receptor superfamily. This receptor is found in a wide array of tissues, including spleen and lungs, and cells such as macrophages, neutrophils, and microglia and is coupled to various specific signaling pathways, depending on where they are expressed [13]. The ALX/FPR2 receptor also responds to resolvins and several peptides, some of which, like annexin-1, are proresolving, while others, such as amyloidogenic peptides, are proinflammatory [14]. Importantly, LXA4 can also bind to additional receptors, including the aryl hydrocarbon receptor AhR [15], the cysteinyl leukotriene receptor (CysLT) [16, 17], the GPR32 receptor [18], and the CB1 cannabinoid receptor [19]. However, LXA4 does not always act as an agonist when bound to these receptors, as it is a partial antagonist of the CysLT receptor [14] and an allosteric signaling enhancer at CB1 cannabinoid receptors [19].

Although LXs are AA-derived eicosanoids, they can be clearly distinguished from the classical proinflammatory prostaglandins, thromboxane, and leukotrienes on the basis of their capacity to trigger a self-limiting response to inflammation when generated by leukocytes. In fact, their formation and functions are directly linked to a change in the phenotype of neutrophils present at the site of inflammation [20]. Once formed at the site of injury, LXs suppress neutrophil recruitment, enhance phagocytosis of apoptotic neutrophils by macrophages, and stimulate the accumulation of a nonphlogistic type of monocytes/macrophages which do not produce proinflammatory mediators [21].

A growing number of studies have demonstrated the roles of LXs as anti-inflammatory and proresolving agents in different animal models of peripheral and central disorders, including cardiovascular diseases, as reviewed by others [6, 10, 22, 23]. Here we will specifically provide an overview of the profile of biological actions of LXs that might be relevant to their potential use as therapeutic agents for inflammatory disorders in the central nervous system (CNS).

## 2. Alzheimer's Disease

Alzheimer's disease (AD) is a devastating neurodegenerative syndrome characterized by drastic and progressive dementia and changes in behavior, allied to accumulation in the brain of extracellular senile plaques composed mainly of amyloid *β* protein (A*β*), intraneuronal neurofibrillary tangles containing hyperphosphorylated tau protein, and chronic neuroinflammation. This disease affects millions of people worldwide, especially late in life, and its causes are incompletely understood [24]. Despite intense efforts, at present AD has no cure and available supportive treatment is far from being efficient. This, in association with the marked increase in life expectancy of the world population, renders the search for more effective treatments of AD one of the greatest challenges in modern medicine.

The role of lipids in AD pathogenesis has been analyzed by several groups, and some studies showed that brains of patients with AD present possible aberrant lipid metabolism [25–27]. The neurodegenerative process in AD is closely related with an inflammatory response in the brain which involves several AA-derived lipid inflammatory mediators [28]. Indeed, a very recent study has revealed that the resolution of inflammation is impaired in the brain of AD patients [29]. The study found that LXA4 levels in postmortem samples of cerebrospinal fluid and hippocampus of AD patients were lower than those of control subjects and that this decrease was correlated with the degree of cognitive deficit and tissue accumulation of tau protein. Conversely, the expression of ALX/FPR2 receptors was clearly greater in AD hippocampal samples.

Intriguingly, amyloid *β* protein (A*β*), one of the major contributors to AD pathogenesis, binds to and activates ALX/FPR2 receptors, but with antagonistic effects [30]. Le and colleagues [30] showed that A*β*_1-42_ exerts chemotactic activity in human leucocytes through ALX/FPR2 receptor activation. Accordingly, another study showed that A*β*, acting via ALX/FPR2 receptors, induces chemotaxis and superoxide production in mouse neutrophils and stimulates cultured murine microglial cells, which strongly suggested its pivotal role in recruitment of microglial cells to senile plaques, induction of oxidative stress, and consequent neuroinflammation in AD [31]. These and other experimental observations clearly establish ALX/FPR2 receptors as pathophysiologically relevant in A*β*-mediated proinflammatory responses in AD [32].

On the other hand, a recent study observed that prolonged twice-daily treatment with the ATL 15-epi-lipoxin A4 (ATLA4) promoted impressive effects in a genetically based murine model of AD [33]. Among the more outstanding findings of the study, ATLA4 downregulated brain production of the proinflammatory mediators TNF-*α*, interleukin-1*β* (IL-1*β*), interferon-*γ*, IL-6, GM-CSF, and RANTES and of MMP-9, all of which are strongly related to AD progression. Conversely, ATLA4 increased brain levels of the anti-inflammatory cytokines IL-10 and TGF-*β*, stimulated the accumulation of alternative microglial cells which, unlike the classical ones, display a nonphlogistic phenotype, and enhanced the clearance of A*β* in CNS. Of note, and in line with earlier observations that A*β* activates the NF*κ*B signaling pathway in the mouse brain [34], ATLA4 treatment also reduced NF*κ*B activation in brain astrocytes (but not in neurons or microglial cells) [34].

In summary, LXA4 and A*β* exert opposing effects at the ALX/FPR2 receptor, and whereas brain LXA4 production is reduced in AD, ALX/FPR2 receptors are overexpressed [29]. At first glance this scenario would strongly favor the strengthening action of A*β* on AD pathogenesis. However, paradoxically, the increased expression of ALX/FPR2 receptors in glial cells during AD should also render the diseased brain more responsive to LXA4, making the treatment with LXs a very interesting option for the AD therapy. Nonetheless, as LXA4 can also interact with additional receptors other than the ALX/FPR2 receptors, the impacts of LXA4 action on such molecular targets on its neuroprotective effects in AD remain to be better characterized. For example, considering that CB1 cannabinoids exert beneficial effects in animal models of AD [35], the fact that LXA4 is an allosteric signaling enhancer at CB1 cannabinoid receptors [19] might be relevant to its potential in AD treatment.

## 3. Stroke

Ischemic stroke is a major cause of morbidity and mortality throughout the world and its outcome depends on the extent of secondary brain damage to the penumbra caused by spreading inflammation [36]. Once a stroke occurs, permeability of the blood-brain barrier (BBB) promptly increases and activates a cascade of inflammatory responses which includes glial activation, neutrophil infiltration, increased expression of selectins and other intercellular adhesion molecules on BBB endothelial cells, as well as an infiltration of immune cells, leading to ischemic brain injury [37–39]. After stroke there is an excessive generation of reactive oxygen species (ROS) that aggravates neuronal death [40, 41]. The changes in BBB permeability seen shortly after the onset of transient or permanent focal ischemia in human patients and in animal stroke models are to a great extent the consequence of increased production of metalloproteinases (MMP), mainly of MMP-9 and MMP-2, by endothelial cells, microglia, and astrocytes [42–51]

As discussed previously, ALX/FPR2 receptors for LXA4 are present in neutrophils, monocytes, macrophages, neural stem cells, and resident cells in the CNS, which render them potential targets for LXA4 in the brain [52–55]. The initial inflammation seen shortly following injury gradually expands to affect a much larger area over several hours to days after a stroke [56, 57]. Brain ischemia rapidly triggers activation of resident glia alongside the recruitment of blood cells [58], and once neutrophils infiltrate the affected area they release phospholipases, proteases, and oxygenated free radicals [56]. Brain unsaturated fatty acids are especially vulnerable to free radical-induced peroxidation. Not surprisingly, therefore, in animal models of stroke the injury can be ameliorated by blocking parts of the inflammatory cascade [59, 60] or limiting neutrophil infiltration at early stages [56, 58, 61].

Several studies have focused on the neuroprotective effects of central LXA4 treatment after stroke [38, 62–64]. Treatment of rats with LXA4 just after transient middle cerebral artery occlusion was found to reduce cerebral infarct volume, neutrophil infiltration, and neuronal apoptosis, and these effects were associated with a better neurological outcome [38]. Importantly, increases in glial cell activation and upregulation in the injured brain of the proinflammatory cytokines, IL-1*β* and TNF-*α*, which are so typical following stroke [65, 66], are also substantially reduced by LXA4 treatment [38, 62]. On the other hand, recovery from stroke has also been associated with upregulation of the anti-inflammatory cytokines, IL-10 and TGF-*β*1 [66, 67], and treatment with LXA4 or BML-111 (the stable synthetic LXA4 analogue 5(S),6(R)-LXA4 methyl ester) has been reported to increase the levels of such cytokines in stroke models involving both the peripheral and the central nervous systems [38, 68]. Such effects of LXA4 in stroke models have been associated with the suppression of NF-*κ*B activation [38, 69, 70], an action which has been clearly evidenced in cultures of epithelial cells and human leukocytes [71, 72]. However, other studies have also implicated the activation of peroxisome proliferator-activated receptor (PPAR) *Υ* [73] and the upregulation of the antioxidant enzyme haeme oxygenase-1 (HO-1) and protein GSH [74] in the anti-inflammatory effects of LXA4 in stroke models.

The MMPs constitute another important target for the beneficial actions of LXA4 in stroke. In this regard, in rats subjected to transient middle cerebral artery occlusion, early postinjury treatment with the LXA4 analogue BML-111 promoted marked reductions in the expression and activity of MMP-9 and MMP-3, as well as an increase in expression of the endogenous MMP inhibitor TIMP-1 in the cortex [64]. This treatment also reduced brain edema, BBB disruption, and infarct size in the cortex, but not in the striatum, which suggests that it selectively attenuated spreading of inflammation throughout the cortex [64]. Moreover, BML-111 treatment dramatically reduced neutrophil infiltration into the brain and microglial cell activation [64]. Inhibition of glial cell activity might be particularly relevant to the anti-inflammatory activity of LXs as ATLA4 markedly reduces LPS-induced reactive oxygen species production in cultured microglial cells [75] and nitric oxide and PGE2 production by iNOS and COX-2 expression in cultured astrocytes [76].

To date only one study has attempted to use antagonists to characterize the receptors mediating the neuroprotective effects of LXA4 in stroke [74]. Of interest, that study showed that combined treatment of rats submitted to middle cerebral artery occlusion with the ALX/FPR2 receptor antagonist Boc-2 (butoxycarbonyl-Phe-Leu-Phe-Leu-Phe) only promoted partial blockade of LXA4-induced reduction in cerebral infarct size and improvement in neurological scores. Moreover, Boc-2 also failed to block LXA4-induced expression of nuclear factor erythroid 2-related factor 2 (Nrf2) and its translocation to the nucleus, as well as that of HO-1 and synthesis of GSH. Indeed, an earlier study had shown that ALX4 activates the Nrf2 signaling pathway in mouse and human macrophages [77]. As this transcription factor coordinates the expression of genes regulated by antioxidant response elements, the Boc-2-resistant Nrf2-dependent effects of LXA4 described by Wu and collaborators [74], that is, increased expression of HO-1 (a redox-sensitive inducible enzyme) and synthesis of GSH (an antioxidant protein), constitute an important ALX/FPR2 receptor-independent mechanism to protect cells from oxidative damage following stroke.

Taken together, the studies reviewed in this section indicate that LXA4, ATLA4, and BML-111 all exert clear cut neuroprotective effects in stroke models. Thus, LXs might hold therapeutic value for the treatment of ischemic stroke. At least part of the neuroprotective effects of LXA4 appear to stem from activation of an Nrf2-GSH/OH-1 signaling pathway.

## 4. Traumatic Brain Injury 

Traumatic brain injury (TBI) is defined as an alteration in brain function or evidence of brain pathology caused by an external force and is related with damage specifically to the brain [78]. An estimated 235,000 Americans are hospitalized annually for nonfatal TBI, and 1.1 million are treated in emergency departments, but, with 50,000 fatal cases every year, TBI is one of the leading causes of mortality among young people [79, 80]. The main causes of TBI include falls, vehicle accidents, assaults, and sports [81].

Surprisingly, the effects of LXA4 treatment in TBI have been largely unexplored. The only study published on this subject so far was carried out in mice subjected to a weight-drop model of TBI, in which the impact was directed to an exposed area of dura mater overlaying the cortex of the left cerebral hemisphere [82]. Injected into the ipsilateral lateral ventricle shortly after trauma, LXA4 was found to reduce BBB permeability, brain edema, and the extent of the lesion. Moreover, the magnitude of the increases in expression of mRNA and protein of the proinflammatory cytokines IL-1*β*, IL-6, and TNF-*α* was significantly smaller in extracts of lesioned cortex taken from LXA4-treated mice relative to TBI controls. The increases in phosphorylated ERK and JNK detected in injured cortex samples at 24 h after TBI were attenuated by LXA4 treatment. Interestingly, although TBI clearly enhanced the activation of cortical astrocytes (as estimated by GFAP immunofluorescence), without apparent change in activity of microglial cells, neither of these parameters were altered by LXA4 treatment. In addition, ALX/FPR2 receptor immunoreactivity seen within the layers of the injured cortex was greatly enhanced in comparison to the sham group and was mostly associated with astrocytes. Indeed, treatment with LXA4 actually increased ALX/FPR2 receptor expression selectively in astrocytes, even if it did not affect astrocyte activation by TBI.

Clearly, this pioneering study of Luo et al. [82] has already disclosed very encouraging actions of LXs in TBI and should stimulate much additional research on this particular topic.

## 5. Neuropathic Pain

The prevalence of chronic pain among the American and European population has been estimated to be around 30%, and about one-fifth of the people who report chronic pain are thought to suffer predominantly neuropathic pain (i.e., about 6% of the total population) [83]. Neuropathic pain is defined as pain resulting from injury to, or dysfunction of, the somatosensory system [84], but this terminology actually encompasses several types of neuropathic pain, most of which are poorly responsive to the drug treatments currently available [83].

Peripheral tissue injury or inflammation commonly triggers reversible changes in the sensory nervous system which enhance the sensitivity to nociceptive pain, a mechanism that protects and ensures proper healing of damaged tissue. By contrast, neuropathic pain is a frequently maladaptive condition resulting from direct injury to the nervous system itself. It is associated with persistent changes in sensitivity of pain pathways to perception of noxious stimuli, so that usually innocuous stimuli evoke pain (allodynia) and responses to noxious stimuli are exaggerated in amplitude (hyperalgesia) and/or duration (hyperpathy), alongside episodes of spontaneous pain [85].

The mechanisms underlying neuropathic pain development are numerous and diverse and frequently involve functional changes to both peripheral and central components of the pain pathways, even when the original injury is inflicted to primary sensory afferents in the periphery [79, 80, 85]. The peripheral sensitization to noxious stimulation is largely due to various alterations in expression and/or activity of ionic channels on nerve fibers, but we will briefly mention just a few of them. Neurotrophins and other mediators generated and released after peripheral nerve injury lower the activation threshold of heat- and acid-sensitive cationic TRPV1 channels and increase their expression not only in injured and uninjured C fibers but also in other primary afferents in which these channels are normally absent. Also, injury to primary sensory afferent fibers induces proliferation and redistribution of many subtypes of voltage-dependent sodium channels (such as Nav1.3, Nav1.7, and Nav1.8) and downregulates the expression and functioning of low voltage-activated and two-pore domain potassium channels. These changes in content and distribution of ion channels in primary afferent fibers are also important to generate ectopic discharges, which are thought to be responsible for neuropathic spontaneous pain. Peripheral nerve injury also induces neuroplastic changes in primary afferent neurons (such as phenotypic switches, collateral sprouting, and synaptic remodeling), augments glutamate release from their central terminals in the dorsal horn of the spinal cord, decreases its local uptake by glial cells, and stimulates spinal second-order nociceptive neurons to overexpress ionotropic NMDA receptors for glutamate. The ensuing potentiation of glutamatergic neurotransmission leads to a central (spinal) sensitization to pain, whereby the repetitive activation of primary afferent fibers causes a progressive increase in the frequency and magnitude of firing of dorsal horn second-order neurons, a phenomenon known as “windup.” Neuropathic pain has also been associated with significant changes in the descending inhibitory and facilitatory controls exerted by supraspinal centers on the input of nociceptive information to the spinal dorsal horn.

Importantly, proinflammatory cytokines, including IL-1*β*, IL-6, and TNF-*α*, are produced peripherally and centrally in response to nerve injury [86]. Therefore, peripheral and central neuroinflammation not only is implicated in the generation and maintenance of chronic inflammatory pain [79, 80] but also is likely to contribute to neuropathic pain [79, 80]. In fact, even if neuropathic and nonneuropathic pains are generally acknowledged to constitute distinct entities, many of the neurotransmitters, neuropeptides, cytokines, and enzymes implicated in both types of pain are the same [83]. In this regard, only a few studies have attempted so far to characterize the effects of LXs and ATLs in models of inflammatory and neuropathic pain.

The first study to assess the effects of LXA4 on pain found that intravenous or intrathecal injections of LXA4, LXB4, or an ATL analogue reduced inflammatory hind paw thermal hyperalgesia induced by carrageenan in rats [54]. The study also reported that spinal astrocytes express ALX/FPR2 receptors and respond to LXA4 with a diminished activation of extracellular signal-regulated kinase and c-Jun N-terminal kinase. Corroborating the view of a regulatory role for LXs in spinal inflammatory nociceptive processing, another study showed that intrathecal LXA4 administration also inhibits the mechanical allodynia and the increase in spinal TNF-*α* levels induced by carrageenan into the hind paw of rats [87].

On the other hand, LXs have also been found to be effective in models of neuropathic pain induced by peripheral nerve injury. In this regard, intrathecal LXA4 injection has been reported to reduce persistently the thermal hyperalgesia and mechanical allodynia which follow chronic unilateral compression of L4 and L5 DRGs in rats [79, 80]. These effects of LXA4 were associated with inhibition, in the compressed DRGs, of the NK-*κ*B signaling pathway and mRNA levels for the proinflammatory cytokines IL-1*β*, IL-6, and TNF-*α*. In addition, repeated intrathecal ATLA4 administration to rats submitted to chronic constriction of sciatic nerve consistently reduced thermal hind paw hyperalgesia and significantly inhibited NALP1 inflammasome activation, caspase-1 cleavage, and IL-1*β* maturation in the spinal cord [79, 80]. Another recent study of the same group reported that the hind paw mechanical allodynia which occurs in the same model was reversed by single intrathecal injections of LXA4 or ATLA4 [79, 80]. The effects of both LXs were abrogated by administration of BOC-2, an ALX/FPR2 receptor antagonist, and most likely involved inhibition of the JAK2/STAT3 signaling pathway and attenuation in the upregulation of mRNA levels for IL-1*β*, IL-6, and TNF-*α* in the spinal cord. Importantly, the neuropathic procedure did not modify the content of ALX4 in neurons and astrocytes of the spinal dorsal horn, and the degree of mechanical allodynia was unaffected by treatment with BOC-2 alone.

Direct lesions to the central nervous system, such as those inflicted by stroke in or traumatic injury to the brain or spinal cord, can also provoke a condition of neuropathic pain known as “central pain” in a significant proportion of patients [88]. The possible effects of LXs in controlling the nociceptive alterations and spontaneous pain associated with these types of injury remain to be estimated, but, from the studies reported in this section, the LXs may constitute a novel means to effectively target pain of both inflammatory and neuropathic pain.

## 6. Conclusions

Over the years, evidence that LXs exert potent neuroprotective and proresolution actions has been consolidated. The identification of their anti-inflammatory properties and effects altered the long-held initial belief that all AA-derived mediators are exclusively proinflammatory, and the evidence accumulated thus far indicates that LXs are powerful proresolving eicosanoids that can profoundly affect several aspects associated with AD, stroke, traumatic brain injury, and neuropathic pain. However, the potential impact of LXs and ATLs in pathological aspects of specific and important conditions, such as spinal cord injury, Parkinson's disease, and Huntington's disease, as well as in other neurodegenerative disorders of the central nervous system is still completely unknown. The studies summarized in the current overview underline the role of LXs in resolution and neuroprotection, but clearly a lot remains to be investigated in relation to the molecular targets of LXs and signaling pathways controlled by them. The development of new potent, selective, and long-acting pharmacological tools targeting different aspects of the LX system would greatly facilitate a better understanding of its importance in modulating diseases of the brain and spinal cord. The evidence available thus far qualifies the LXs as potent agonists for neuromodulation, neurological protection, and resolution of the diseased CNS and highlights the potential of treatments based on LXs in the management of neurodegenerative diseases affecting the brain and spinal cord.
